# Large-for-gestational-age phenotypes and obesity risk in adulthood: a study of 195,936 women

**DOI:** 10.1038/s41598-020-58827-5

**Published:** 2020-02-07

**Authors:** José G. B. Derraik, Sarah E. Maessen, John D. Gibbins, Wayne S. Cutfield, Maria Lundgren, Fredrik Ahlsson

**Affiliations:** 1 Liggins Institute, University of Auckland, Auckland, New Zealand; 2 A Better Start – National Science Challenge, University of Auckland, Auckland, New Zealand; 3 Department of Women’s and Children’s Health, Uppsala University, Uppsala, Sweden; 4 Department of Endocrinology, Children’s Hospital, Zhejiang University School of Medicine, Hangzhou, P.R. China

**Keywords:** Obesity, Epidemiology

## Abstract

While there is evidence that being born large-for-gestational-age (LGA) is associated with an increased risk of obesity later in life, the data are conflicting. Thus, we aimed to examine the associations between proportionality at birth and later obesity risk in adulthood. This was a retrospective study using data recorded in the Swedish Birth Register. Anthropometry in adulthood was assessed in 195,936 pregnant women at 10–12 weeks of gestation. All women were born at term (37–41 weeks of gestation). LGA was defined as birth weight and/or length ≥2.0 SDS. Women were separated into four groups: appropriate-for-gestational-age according to both weight and length (AGA – reference group; n = 183,662), LGA by weight only (n = 4,026), LGA by length only (n = 5,465), and LGA by both weight and length (n = 2,783). Women born LGA based on length, weight, or both had BMI 0.12, 1.16, and 1.08 kg/m^2^ greater than women born AGA, respectively. The adjusted relative risk (aRR) of obesity was 1.50 times higher for those born LGA by weight and 1.51 times for LGA by both weight and height. Length at birth was not associated with obesity risk. Similarly, women born LGA by ponderal index had BMI 1.0 kg/m^2^ greater and an aRR of obesity 1.39 times higher than those born AGA. Swedish women born LGA by weight or ponderal index had an increased risk of obesity in adulthood, irrespective of their birth length. Thus, increased risk of adult obesity seems to be identifiable from birth weight and ignoring proportionality.

## Introduction

Obesity is a rapidly growing problem, and it was estimated that in 2015 there were 107.7 million children and 603.7 million adults with obesity worldwide^1^. Furthermore, the prevalence of obesity doubled from 1980 to 2015 in over 70 countries^1^. This is particularly concerning considering the associations between obesity and many chronic diseases, such as diabetes, cardiovascular disease^2^, and some cancers^3^, as well as an increased risk of mortality^4^. In addition, a high BMI in adolescence is associated with a high prevalence of cardiovascular death in adulthood^5,6^. Thus, it is imperative that we improve our understanding of the factors that promote obesity from an early age, so that we may identify those at greatest risk and develop strategies to prevent the development of obesity.

Several studies have previously shown that being born large-for-gestational-age (LGA) is a predictor of obesity in adulthood^7–10^. In many countries, the prevalence of infants born LGA has increased over the last few decades^11–15^. The proportion of newborns weighing >4000 g ranges from less than 1% to 14.9% in developing countries^13^, and is as high as 20% in Nordic countries^12^. However, the long-term impact of being born LGA is not completely understood, with one study finding that LGA children were at greater risk of developing metabolic syndrome at aged 6–11 only if their mothers had gestational diabetes mellitus^13^. In addition, if their mothers did not have gestational diabetes then there was little difference in the rates of obesity at ages 6–11 for those born LGA compared with those born apropriate-for-gestational-age (AGA)^13^. Similarly, another study found no difference in the risk of being overweight/obesity at ages 15–20 years if born LGA compared with those born AGA^14^.

Less is known regarding the association between being born long for gestational age or being born proportionately large (long and heavy) and the risks of developing obesity, as most studies have focused on offspring weight alone^15^. However, a lower ponderal index (weight in relation to length) at birth has been linked to lower total body fat in childhood^16^. It has been also suggested that proportionally heavier infants may be at greater risk of poor cardiovascular and metabolic outcomes later in life^17^. Only two studies have examined whether proportionality at birth was related to obesity risk beyond childhood, and only one included female participants. The first study examined the association between birth weight and length and obesity risk in young male conscripts^18^. They reported that a high birth weight was associated with an increased risk of obesity at age 18 years, and this association remained even when the increased weight was proportional to length. The second study reported that both higher weight and ponderal index at birth were associated with an increased risk of obesity for men, but not for women^19^. However, their conclusion that birth weight was unrelated to risk of obesity in women contradicts at least one previous study^9^. Additionally, two studies report that being born long for gestational age alone does not increase the risk of obesity in adults^20,21^. Overall, there are few studies on the associations between birth proportionality and long-term health outcomes. This is particularly true for studies reporting on outcomes in women, despite sex-specific differences being frequently identified in the long-term effects of early life events^22^.

As such, the role that birth weight and proportionality play in determining the risk of obesity in later life for females is unclear. Thus, in the present study we aimed to assess the risk of adult obesity among a large cohort of Swedish women who were born too large by weight and/or length in comparison to those born AGA.

## Methods

### Ethics

Ethics approval for this study was granted by the Regional Ethical Review Board in Uppsala, Sweden (Dnr2011/141). This investigation was carried out in accordance with approved national and international guidelines for medical research. Informed consent was not required as this is a register-based study on anonymized data where participants were neither identified nor contacted. As explained by Ludvigsson *et al*.^23^, informed consent is generally not required for large registry-based studies in Sweden and other Nordic countries, where it is assumed that participants do not object to registry-based research, as long as the study has been approved by the relevant ethics committee. The authors add that this is “part of the informal contract between the individual and the state (…), given that health care is traditionally virtually free of charge (…), and registry-based data are maintained for the purpose of health care quality improvement and national statistics”^23^.

### Swedish birth register

This was a retrospective study using data recorded in the Swedish Birth Register. This Register records data on more than 99% of births in the country, and for the study period it had a low error rate for the main parameters of relevance, such as birth weight, date of last menstrual period, birth order, and classification as singleton or multiple^24^. Information is prospectively collected during pregnancy from the first antenatal visit and subsequently forwarded to the Birth Register.

### Study population

We examined data recorded on 303,301 pregnant women (born in 1973–1988 in Sweden) during their first antenatal visit (mostly 10–12 weeks of gestation), who gave birth in 1991–2009 and were aged ≥18 years. For women with two or more pregnancies in the study period, data were only included for the first recorded pregnancy. Exclusion criteria were non-Nordic ethnicity, extremely short stature (≤130 cm), being born small-for-gestational-age [SGA; < −2 standard deviation scores (SDS) below the Swedish population mean for birth weight and/or birth length^25^], presence of congenital malformations (ICD-9 740–759 and ICD-10 Q0–Q99), preterm birth (<37 weeks of gestation)^26,27^, and post-term birth (≥42 weeks of gestation)^28–30^.

### Measurements

Birth weight and birth length were transformed to SDS as per Niklasson *et al*.^25^, with LGA defined as being ≥2.0 SDS heavier and/or longer than the respective population means. Ponderal index was calculated as per Röhrer’s formula:

$${\rm{p}}{\rm{o}}{\rm{n}}{\rm{d}}{\rm{e}}{\rm{r}}{\rm{a}}{\rm{l}}\,{\rm{i}}{\rm{n}}{\rm{d}}{\rm{e}}{\rm{x}}\,({\rm{g}}/{{\rm{c}}{\rm{m}}}^{3})=\frac{100\,\ast \,{\rm{w}}{\rm{e}}{\rm{i}}{\rm{g}}{\rm{h}}{\rm{t}}}{{\rm{l}}{\rm{e}}{\rm{n}}{\rm{g}}{\rm{t}}{{\rm{h}}}^{3}}$$

LGA by ponderal index was defined as ≥2.0 SDS above our population mean. Individuals with implausible values (SDS < −5.0 or >5.0) were excluded from analyses.

Weight was measured by a midwife, while height was self-reported in most cases. Gestational age of the women at their birth (extracted from the Swedish Birth Register) was estimated from the date of the last menstrual period for the majority of participants, otherwise estimates were based on ultrasound scans. Underweight was defined as body mass index (BMI) <18.5 kg/m^2^; normal weight ≥18.5 and <25 kg/m^2^; overweight ≥25 and <30 kg/m^2^; obesity class I ≥30 and <35 kg/m^2^; obesity class II ≥35 and <40 kg/m^2^; obesity class III ≥40 kg/m^2^.

### Statistical analyses

Age and demographic data at birth were compared between groups using univariate general linear regression models. The comparison group for all analyses were women who were born AGA for both weight and length. Anthropometric differences in adulthood were examined with generalized linear regression models. All adjusted models included the smoking habit of the woman’s mother during pregnancy^31^, birth order^32,33^, and year of birth (to account for population-wide trends). Weight and BMI models also included current regular smoking (pre-pregnancy and/or during pregnancy) and age, while the “weight [ht adj]” model also adjusted for current height.

Logistic regression models were run to evaluate binary outcomes (i.e. likelihood of overweight and/or obesity in adulthood), with adjusted models accounting for the smoking habit of the woman’s mother during pregnancy, birth order, year of birth, current regular smoking (pre-pregnancy and/or during pregnancy), and age. Analyses were performed in SAS v9.4 (SAS Institute, Cary, USA) and SPSS v24 (IBM Corp, Armonk, NY, USA). All tests were two-tailed, with significance level adjusted for multiple comparisons using the Tukey-Kramer method^34^.

## Results

**Figure 1:**
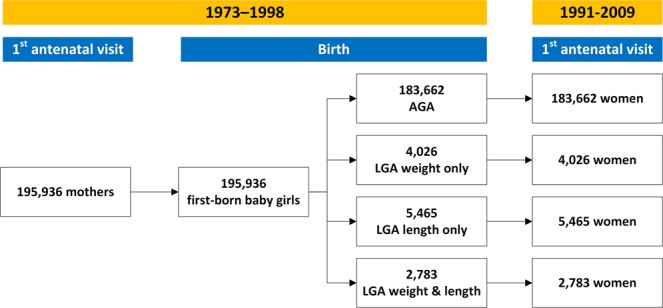
Diagram showing studied participants with data extracted from the Swedish Birth Register. LGA, large-for-gestational-age.

From the initial population of 303,301, we studied 195,936 women who met inclusion criteria (Fig. 1; Supplementary Tables 1 and 2). The study participants had a mean age of 26.0 years (range 18 to 36 years; Supplementary Fig. 1). A total of 5,465 women were born LGA by length only (2.8%), 4,026 by weight only (2.1%), and 2,783 (1.4%) by both (Table 1). At birth, the groups had marked anthropometric differences in all parameters measured (Table 1). Of note, the likelihood of the women having been born LGA by weight only or LGA by both weight and length was progressively higher with their mother’s increasing BMI early in pregnancy (Table 2).

**Table 1:** Parameters recorded at birth for women born appropriate-for-gestational-age (AGA) according to both weight and length and those born large-for-gestational-age (LGA) by weight, length, or both.

	AGA	LGA Both	LGA Weight only	LGA Length only
n	183,662 (93.7%)	2,783 (1.4%)	4,026 (2.1%)	5,465 (2.8%)
Birth weight (g)	3,422 ± 395^A^	4,601 ± 290^B^	4,412 ± 265^C^	4,002 ± 278^D^
Birth weight SDS	0.10 ± 0.84^A^	2.57 ± 0.48^B^	2.36 ± 0.36^C^	1.22 ± 0.56^D^
Birth length (cm)	49.9 ± 1.7^A^	54.5 ± 1.0^B^	51.9 ± 1.1^C^	54.2 ± 0.70^D^
Birth length SDS	0.17 ± 0.85^A^	2.61 ± 0.49^B^	1.33 ± 0.52^C^	2.38 ± 0.38^D^
Ponderal index (g/cm^3^)	2.74 ± 0.22^A^	2.85 ± 0.18^B^	3.16 ± 0.23^C^	2.51 ± 0.18^D^
Ponderal index SDS	−0.02 ± 0.96^A^	0.44 ± 0.75^B^	1.77 ± 0.99^C^	−0.99 ± 0.76^D^
Gestational age (weeks)	39.8 ± 1.9^A^	40.1 ± 1.9^B^	39.8 ± 2.0^C^	40.1 ± 1.9^D^

Data are means ± standard deviations.

SDS, standard deviation scores.

Different superscript letters indicate groups that are statistically significant from each other after Tukey-Kramer’s adjustment for multiple comparisons.

**Table 2:** Associations between the BMI status of the woman’s mother early in pregnancy and the woman’s phenotype at birth.

		Woman’s phenotype at birth			
		AGA	LGA Both	LGA Weight only	LGA Length only
n		183,662 (93.7%)	2,783 (1.4%)	4,026 (2.1%)	5,465 (2.8%)
Mother’s BMI status^**†**^	Underweight	2,166 (97.4%)	12 (0.5%)	25 (1.1%)	21 (0.9%)
	Normal weight	16,995 (94.4%)	228 (1.3%)	361 (2.0%)	411 (2.3%)
	Overweight	2,898 (88.8%)	94 (2.9%)	154 (4.7%)	119 (3.9%)
	Obesity	631 (87.3%)	33 (4.6%)	40 (5.5%)	19 (2.9%)

^†^Recorded at the woman’s mother’s first antenatal visit.

Data are n (%).

AGA, appropriate-for-gestational-age according to both weight and length; BMI, body mass index; LGA, large-for-gestational-age.

Underweight BMI <18.5 kg/m^2^; normal weight ≥18.5 and <25 kg/m^2^; overweight ≥25 and <30 kg/m^2^; and obesity ≥30 kg/m^2^.

### Weight vs length

There were marked anthropometric differences among women born LGA (Table 3). In adulthood, women in all three LGA groups were taller and heavier than those born AGA (Table 3). Women born LGA by weight or both (weight and length) had BMI that were 1.16 kg/m^2^ and 1.08 kg/m^2^ greater than those born AGA, respectively; however, the difference was only 0.12 kg/m^2^ for women LGA by length only (Table 3). These results were largely unchanged after adjustment for confounders.

**Table 3:** Anthropometric data of women born appropriate-for-gestational-age or large-for-gestational-age by weight, length or both.

		AGA	LGA Both	LGA Weight only	LGA Length only
n		183,662	2,783	4,026	5,465
Age (years)		26.0 ± 4.0	25.9 ± 3.9	25.8 ± 4.0	26.2 ± 3.9
Unadjusted	Height (cm)	167.0 (167.0–167.0)^A^	172.4 (172.2–172.6)^D^	169.8 (169.7–170.0)^B^	171.8 (171.6–171.9)^C^
	Weight (kg)	67.18 (67.12–67.24)^A^	74.79 (74.31–75.26)^D^	72.80 (72.41–73.19)^B^	71.40 (71.07–71.74)^C^
	BMI (kg/m^2^)	24.07 (24.05–24.09)^A^	25.16 (25.00–25.32)^B^	25.24 (25.10–25.37)^B^	24.19 (24.08–24.30)^A^
	Underweight	5,353 (2.9%)	34 (1.2%)	51 (1.3%)	109 (2.0%)
	Normal weight	121,182 (66.0%)	1619 (58.2%)	2338 (58.1%)	3604 (65.9%)
	Overweight	39,797 (21.7%)	730 (26.2%)	1056 (26.2%)	1240 (22.7%)
	Obesity class I	12,233 (6.7%)	268 (9.6%)	366 (9.1%)	370 (6.8%)
	Obesity class II	3,778 (2.1%)	102 (3.7%)	165 (4.1%)	101 (1.8%)
	Obesity class III	1,319 (0.7%)	30 (1.1%)	50 (1.2%)	41 (0.8%)
Adjusted	Height (cm)	167.0 (167.0–167.1)^A^	172.4 (172.2–172.7)^D^	169.9 (169.7–170.1)^B^	171.8 (171.6–171.9)^C^
	Weight (kg)	67.75 (67.68–67.82)^A^	75.48 (74.97–75.98)^D^	73.36(72.94–73.78)^B^	72.05 (71.68–72.41)^C^
	Weight [ht adj] (kg)	67.96 (67.90–68.03)^A^	71.89 (71.41–72.37)^B^	71.58 (71.18–71.98)^B^	68.95 (68.61–69.30)^C^
	BMI (kg/m^2^)	24.27 (24.24–24.29)^A^	25.37 (25.20–25.54)^B^	25.41 (25.27–25.55)^B^	24.41 (24.28–24.53)^A^

Data were recorded early in pregnancy (mostly 10–12 weeks) in 1991–2009 among 195,936 women who were born in Sweden in 1973–1988.

AGA, appropriate-for-gestational-age according to both weight and length; BMI, body mass index; LGA, large-for-gestational-age.

Underweight BMI <18.5 kg/m^2^; normal weight ≥18.5 and <25 kg/m^2^; overweight ≥25 and <30 kg/m^2^; obesity class I ≥30 and <35 kg/m^2^; obesity class II ≥35 and <40 kg/m^2^; and obesity class III ≥40 kg/m^2^.

Age data are means ± standard deviations; BMI category data are n (%); other data are means and 95% confidence intervals. Different superscript letters indicate groups that are statistically significant from each other after Tukey-Kramer’s adjustment for multiple comparisons. Data were analysed using generalized linear regression models. All adjusted models included as factors the smoking habit of the woman’s mother during pregnancy, birth order, and year of birth. Weight and BMI models also included current regular smoking (pre-pregnancy and/or during pregnancy) and age, while the “weight [ht adj]” model also adjusted for current height.

Notably, after the differences in height were accounted for, women born LGA by weight or both were 3.6 kg and 3.9 kg heavier as adults than AGA women, while those LGA by length only were 1.0 kg heavier (Table 3). In addition, a greater proportion of women born LGA by weight or both were overweight or had obesity of any class compared to peers born AGA (Table 3). Conversely, the proportions of women who were overweight or had obesity was similar among those born AGA or LGA by length alone (Table 3).

**Figure 2:**
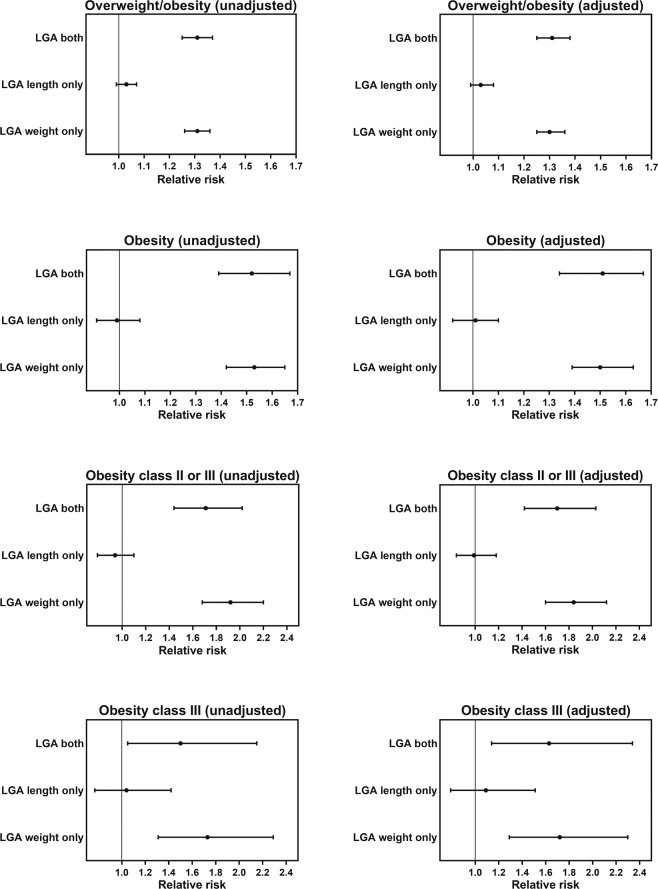
Risk of overweight and/or obesity early in pregnancy among Swedish women born large-for-gestational-age (LGA) according to weight and/or length. Women were born in Sweden in 1973–1988, and body mass index (BMI) data were recorded in 1991–2009 at a mean age of 26.0 years. The reference group were women who were born appropriate-for-gestational age according to both weight and length. Data are unadjusted and adjusted relative risks with respective 95% confidence intervals of overweight/obesity (BMI ≥25 kg/m^2^), obesity (BMI ≥30 kg/m^2^), obesity class II/II (BMI ≥35 kg/m^2^), or obesity class III (BMI ≥40 kg/m^2^) for women born LGA age by length, weight, or both. Adjusted relative risks accounted for the smoking habit of the woman’s mother during pregnancy, birth order, year of birth, current regular smoking, and age.

The aRR of obesity was 1.50 (95% CI 1.39, 1.63) and 1.51 (1.37, 1.67) among women born LGA by weight or both, respectively, compared with those in the AGA group (Fig. 2). The risk of having BMI at the upper end of the spectrum was even greater, and the aRR of obesity class II/III and class III in women born LGA by weight was 1.84 (95% CI 1.60, 2.12) and 1.72 (1.29, 2.30), respectively, while in those born LGA by both weight and length it was 1.70 (1.42, 2.03) and 1.63 (1.14, 2.34) (Fig. 2). In contrast, there was no increase in the risk of overweight or obesity at any level of severity among women born LGA by length only (Fig. 2).

### Ponderal index

**Figure 3:**
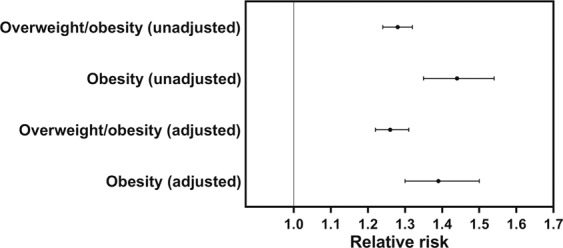
Risk of overweight and/or obesity among women born large-for-gestational-age by ponderal index compared with those born appropriate-for-gestational-age. Women were born in Sweden in 1973–1988, and BMI data were recorded in 1991–2009 at a mean age of 26.0 years. Adjusted relative risks accounted for the smoking habit of the woman’s mother during pregnancy, birth order, year of birth, current regular smoking (pre-pregnancy and/or during pregnancy), and age. Overweight/obesity: body mass index (BMI) ≥ 25 kg/m^2^; and obesity: BMI ≥30 kg/m^2^.

A total of 5,546 women (2.8%) were born LGA by ponderal index, who were 1.22 SDS heavier and 0.48 SDS shorter at birth than those born AGA (Supplementary Table 3). In adulthood, women born LGA by ponderal index were on average 1.1 cm shorter and nearly 1.8 kg heavier, with a BMI that was 1.0 kg/m^2^ greater than those born AGA (Table 4). These differences were largely unchanged in adjusted analyses, except that when their heights were accounted for, the weight difference between the two groups increased to 2.5 kg (Table 4). As a result, the aRR of obesity in adulthood was 1.39 times greater in women born LGA by ponderal index compared with the women born AGA (95% CI 1.30–1.50; Fig. 3).

**Table 4:** Anthropometric data among women born large-for-gestational-age (LGA) or not according to ponderal index at birth.

		AGA by ponderal index	LGA by ponderal index
n		190,214 (97.2%)	5,546 (2.8%)
Age (years)		26.0 ± 4.0	25.6 ± 3.9
Unadjusted	Height (cm)	167.3 (167.3–167.3)	166.2 (166.1–166.4)****
	Weight (kg)	67.47 (67.41–67.53)	69.31 (68.97–69.64)****
	BMI (kg/m^2^)	24.09 (24.07–24.11)	25.07 (24.95–25.18)****
	Underweight	5,447 (2.9%)	97 (1.7%)
	Normal weight	125,389 (65.9%)	3,237 (58.4%)
	Overweight	41,335 (21.7%)	1,453 (26.2%)
	Obesity	18,043 (9.5%)	759 (13.7%)
	Overweight/obesity	59,378 (31.2%)	2,212 (39.9%)
Adjusted	Height (cm)	167.3 (167.3–167.4)	166.3 (166.1–166.4)****
	Weight (kg)	68.04 (67.85–68.22)	69.80 (69.44–70.15)****
	Weight [ht adj] (kg)	68.05 (67.98–68.11)	70.54 (70.20–70.88)****
	BMI (kg/m^2^)	24.28 (24.26–24.30)	25.22 (25.10–25.34)****

AGA, appropriate-for-gestational-age; BMI, body mass index; underweight: BMI <18.5 kg/m^2^; normal weight: BMI ≥18.5 kg/m^2^ and <25 kg/m^2^; overweight: BMI ≥25 kg/m^2^ and <30 kg/m^2^; overweight/obesity: BMI ≥25 kg/m^2^; and obesity: BMI ≥ 30 kg/m^2^.

Age data are means ± standard deviation; body mass index (BMI) category data are n (%); other data are means and 95% confidence intervals.

****p < 0.0001 for comparisons between groups.

Data were analysed using generalized linear regression models. All adjusted models included as factors the smoking habit of the woman’s mother during pregnancy, birth order, and year of birth. Weight and BMI models also included current regular smoking (pre-pregnancy and/or during pregnancy) and age, while the “weight [ht adj]” model also adjusted for current height.

### Temporal trends

**Figure 4:**
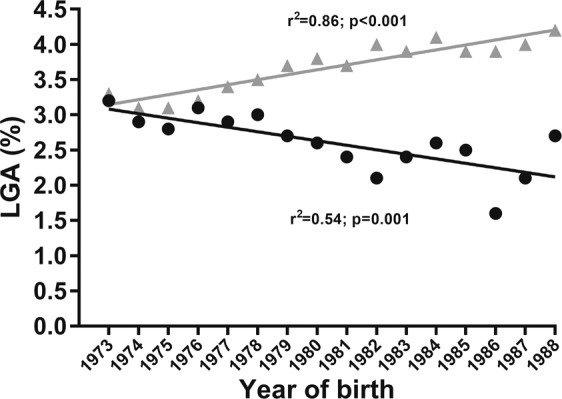
Rates of large-for-gestational-age (LGA) phenotypes at birth amongst the Swedish women in the study. Black circles represent girls born LGA according to length only, while gray triangles are girls born LGA according to weight or both.

Over the 15-year recruitment period for this study, there was a progressive increase in the rates of girls born LGA by weight only or both (i.e. length and weight) (Fig. 4). In contrast, there was a steady reduction in the prevalence of girls born LGA by length only (Fig. 4).

## Discussion

This study found that, on average, Swedish women born LGA by weight were 50% more likely to have obesity in adulthood, even if they were proportionally longer at birth. Women who were classified as LGA by ponderal index (i.e. were heavier and shorter than those born AGA) were also more likely to have obesity in adulthood. Women born LGA by length alone were not at increased risk of obesity. Of note there was a change in the distribution of LGA phenotype over the study period, with more girls born LGA by weight and fewer LGA by length only. Our data indicate that the most accurate way to identify those at greater risk of obesity in adulthood are those who are born LGA based on weight, and this association was not mitigated if the babies were also born proportionally longer. This is particularly important given the proportional increase observed in the rate of girls born LGA by weight and the corresponding decrease in the annual rates of those born LGA by length only.

Our findings on Swedish women corroborate a previous study on 18-year-old male conscripts in Sweden^18^. In that study, infants who were above the 95th percentile for birth weight had a higher risk of being overweight or having obesity at age 18 years. Similarly to our study, the increased risk was observed among heavy infants who had a normal birth length as well as those who were above the 95^th^ percentile at birth for both weight and length^18^. They found that infants who were born above the 95^th^ percentile for length alone did not have an increased risk of overweight or obesity later in life^18^.

Our results are also consistent with the well-established association between high birth weight and an increased risk of obesity in later life^7–10,35^. Conversely, our results differ to those reported by Eriksson *et al*. who found no relationship between high birth weight or ponderal index and increased risk of obesity in adulthood for Finnish women^19^. However, their study differs on three key aspects: a) the use of self-reported heights and weights that often lead to underestimation of BMI among women^36^; b) a much smaller sample of 2,107 women, with fewer than 1% falling into their highest birth weight category; and c) participants born in the 1920s and early 1930s who were likely exposed to very different *in utero* and postnatal environments that could have differentially affected their long-term obesity risk.

Similarly to our findings, Rasmussen & Johansson found that a higher ponderal index at birth was associated with increased BMI in adulthood^18^. Ponderal index at birth has been shown to predict total body fat in childhood better than birth weight alone^16^. However, in our study, we found that being too heavy at birth was associated with a greater risk of adult obesity than being born too large by ponderal index. This may be because ponderal index has only a weak association with body fat in neonates (r^2^ = 0.15; p = 0.001), and is not a direct proxy for corpulence as it is often assumed to be^37^. Thus, it is possible that the amount of fat mass at birth is a better predictor of adiposity later in life, rather than the ratio between weight and length at birth.

Notably, it is important to highlight that being born LGA is not the cause of adult obesity *per se*; rather, it is a marker of events/stressors that occur before and/or during pregnancy. Babies are born LGA likely due to excessive nutrition *in utero*, which are associated with alterations in the developing fetus that will predispose them to obesity postnatally^38^. Both maternal obesity and gestational diabetes mellitus are associated with increased adiposity and greater BMI at birth^39,40^. Fetal secretion of insulin in response to increased maternal glucose levels drives fetal growth, and increased maternal nutrient supply to the fetus will result in higher insulin levels and greater adiposity, and subsequently to developmental programming of fetal adipose tissue^41^. In light of the increasing rates of obesity among women of reproductive age worldwide, preventive measures to address this problem and reduce the incidence of babies born in the upper end of the birth weight spectrum are likely to be beneficial in the long-term. These preventative measures should include appropriate lifestyle advice to those planning to conceive as well as women who are already pregnant^15^. For example, women with obesity may lower the risk of having a baby born LGA by limiting their weight gain during pregnancy^15^. Interventions aiming at assisting women to improve their health before pregnancy would have beneficial effects not only for their health, but also for future generations by breaking the intergenerational cycle of obesity^42^.

For babies already born LGA by weight (and thus with an increased risk of obesity later in life), there are measures that may prevent the onset of obesity. For example, two meta-analyses found that breastfeeding was associated with lower risk of overweight and/or obesity in childhood, and both observed a dose-dependent association, with the protective effect increasing with greater duration of breastfeeding^43^. Interestingly, Goetz *et al*.^44^ found that children born LGA who were heavier at 7–12 months were more likely to have received proportionally less breast milk feedings from birth to 6 months compared to those born LGA who had normal weight at 7–12 months. Notably, there is evidence that even simple measures as reducing bottle size for those babies receiving formula may help prevent excessive weight gain, and consequently reduce the risk of obesity later in life^45,46^. In addition, fostering improvements in sleep^47^, diet, and physical activity early on the child’s life would likely play a role in reducing the risk of developing obesity.

A limitation of our study was the lack of information on the post-natal environment, particularly regarding factors such as socioeconomic status and education; for example, the odds of obesity in Swedish male conscripts were 2.3 times higher amongst the offspring of mothers with lower levels of education^18^. Another limitation was the absence of data on pregnancy complications, particularly gestational diabetes. However, strengths of our study include our large sample size and our somewhat homogeneous population, with the latter likely to mitigate the potential effects of ethnicity on study outcomes. Importantly, our study accounted for maternal anthropometry, which is arguably the strongest predictor of offspring anthropometry and obesity risk, both at birth and in adulthood^48–50^. Of note, while we did not have data on paternal anthropometry, we have recently showed this to have no association with the likelihood of an LGA baby when maternal anthropometry was accounted for^50^. In addition, our multivariable models also adjusted for the smoking habit of the woman’s mother during pregnancy, which is also strongly associated with obesity risk in their adult daughters in Sweden^31^.

## Conclusions

We showed that the LGA phenotype has a marked association with the risk of adult obesity. LGA by length is more likely to be genetically rather than nutritionally determined, and is therefore not associated with an increased risk of obesity in the long-term. Conversely, being LGA by weight is a major risk factor for adult obesity, and this holds true even if they were also born long for gestational age. When assessing the long-term health risks associated with being born LGA, it is therefore important to consider the specific phenotype. Our findings illustrate that it is essential that we address the problem of obesity early, and the best strategy to prevent adverse health outcomes in both shortterm and longterm is prevention. Interventions should aim to address the obesity issue before women decide to become pregnant, particularly through the promotion of better lifestyle choices, which would in turn likely reduce the risk of having an LGA infant that is too heavy. However, for those already born too heavy, understanding the risk is a tool to encourage behavioural changes that would help reduce the likelihood of adverse health outcomes, such as the development of obesity throughout the life span. These could include early feeding practices (e.g. breastfeeding) and lifestyle changes (such as modification of dietary habits and increased levels of physical activity).

## Supplementary information

Supplementary File.

## Data Availability

Data used in this study were obtained from the Swedish Medical Birth Register. While these data cannot be made publicly available, they can be accessed upon request to the Swedish National Board of Health and Welfare, pending approval by the appropriate ethics committee. Information on the Birth Register and persons to contact for queries regarding access are available in English from: https://www.socialstyrelsen.se/en/statistics-and-data/registers/register-information/the-swedish-medical-birth-register/.
