# Supplementary material for: Large-for-gestational-age phenotypes and obesity risk in adulthood: a study of 195,936 women
Source: Sci Rep. 2020 Feb 7;10:2157. doi: 10.1038/s41598-020-58827-5 (PMC7005699; doi:10.1038/s41598-020-58827-5)
Supplement: Supplementary file 1 — Supplementary File. [file 41598_2020_58827_MOESM1_ESM.pdf]

Supplementary File

Large-for-gestational-age phenotypes and obesity risk in adulthood: a study of 195,936 women

José G B Derraik[1,2,3,4*], Sarah E Maessen[1], John D Gibbins[1], Wayne S Cutfield[1,2,4], Maria Lundgren[3], Fredrik Ahlsson[3*]

[1] Liggins Institute, University of Auckland, Auckland, New Zealand

[2] A Better Start – National Science Challenge, University of Auckland, Auckland, New Zealand

[3] Department of Women's and Children's Health, Uppsala University, Uppsala, Sweden

[4] Department of Endocrinology, Children's Hospital, Zhejiang University School of Medicine.

*Authors for correspondence:

Dr José Derraik – jderraik@gmail.com

Associate Professor Fredrik Ahlsson – fredrik.ahlsson@kbh.uu.se

Supplementary Table 1

Reasons for exclusion of participants from the study and the respective *n*.

**Original *n***		303,301
**Exclusion criteria**	Missing height data	18,826
	Missing weight data	16,263
	Missing birth weight	441
	Missing birth length	640
	Born with any malformation	10,627
	Twins/multiples	2,280
	Born preterm (<37 weeks of gestation)	10,884
	Born post-term (≥42 weeks of gestation)	31,111
	Born from non-Nordic mothers	8,012
	Too short (<130 cm tall)	15
	Aged <18 years at antenatal visit	2,651
	Born small-for-gestational-age by weight	3,881
	Born small-for-gestational-age by length	1,707
	Implausible birth weight or birth length*	27
**Included *n***		**195,936**

* Defined as a birth weight and/or length ≥5 standard deviation scores.

Supplementary Table 2

The distribution of our study participants according to their year of birth.

Year of birth	Frequency	Percent	Cumulative Percent
1973	23,726	12.1	12.1
1974	23,871	12.2	24.3
1975	21,709	11.1	35.4
1976	19,600	10.0	45.4
1977	18,112	9.2	54.6
1978	16,149	8.2	62.9
1979	15,006	7.7	70.5
1980	12,742	6.5	77.0
1981	10,541	5.4	82.4
1982	8,664	4.4	86.8
1983	7,075	3.6	90.4
1984	5,657	2.9	93.3
1985	4,710	2.4	95.7
1986	3,833	2.0	97.7
1987	2,697	1.4	99.1
1988	1,844	0.9	100.0
Total	195,936	100.0	

Supplementary Table 3

Parameters recorded at birth for women born in Sweden in 1973–1988 who were large-for-gestational-age (LGA) or not according to ponderal index at birth.

	AGA	LGA BY PONDERAL INDEX	*P*-VALUE
n	190,214 (97.2%)	5,546 (2.8%)	
**Birth weight (g)**	3,458 ± 433	4,036 ± 444	<0.0001
**Birth weight SDS**	0.18 ± 0.92	1.40 ± 0.90	<0.0001
**Birth length (cm)**	50.2 ± 1.9	49.4 ± 1.8	<0.0001
**Birth length SDS**	0.30 ± 0.96	-0.18 ± 0.93	<0.0001
**Ponderal index (g/cm^3)**	2.73 ± 0.21	3.33 ± 0.11	<0.0001
**Ponderal index SDS**	-0.08 ± 0.90	2.51 ± 0.49	<0.0001
**Gestational age (weeks)**	39.6 ± 1.1	39.7 ± 1.0	<0.0001

AGA, appropriate-for-gestational-age; SDS, standard deviation score.
Data are means ± standard deviations or *n* (%).

Supplementary Figure 1

Histogram showing the age distribution of study participants, as recorded at their first antenatal visit.

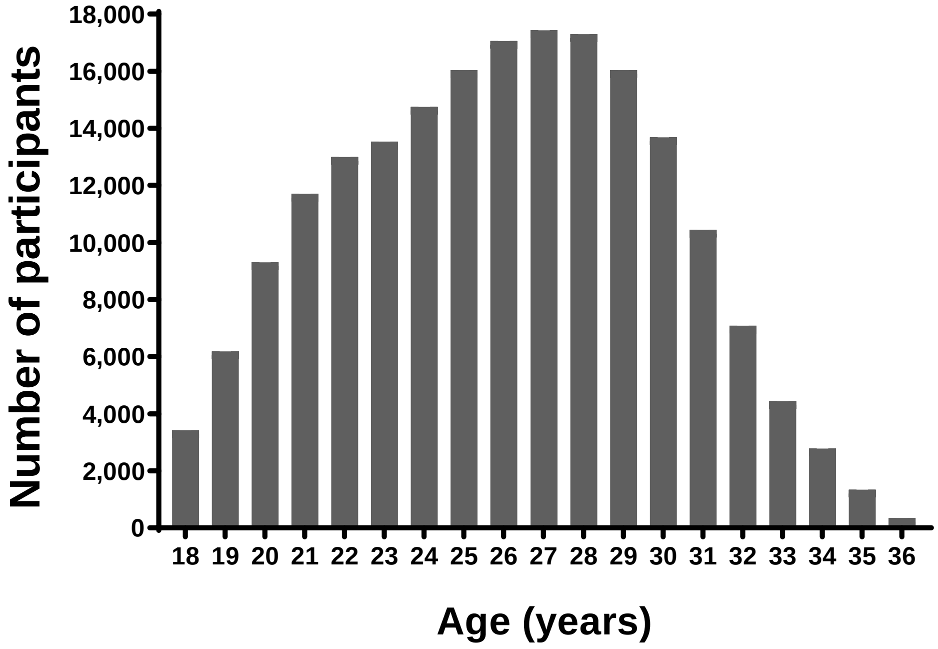

Derraik JGB, Maessen SE, Gibbins JD, Cutfield WS, Lundgren M, Ahlsson F. Large-for-gestational-age phenotypes and obesity risk in adulthood: a study on 195,936 Swedish women. Scientific Reports.
